# Trend in the incidence rates of accidents with venomous animals in children and adolescents in Brazil (2007–2019)

**DOI:** 10.1590/1984-0462/2023/41/2021272

**Published:** 2022-07-06

**Authors:** Thais Cláudia Roma de Oliveira Konstantyner, Camila Bertini Martins, Aécio Flávio Teixeira de Góis, Braian Valério Cassiano de Castro, Tulio Konstantyner

**Affiliations:** a Universidade Federal de São Paulo, São Paulo, SP, Brazil.; b Faculdade de Ciências Médicas, Santa Casa de São Paulo, São Paulo, SP, Brazil.

**Keywords:** Animals, poisonous, Public health surveillance, Epidemiological monitoring, Incidence, Accidents, Animais venenosos, Vigilância em saúde pública, Vigilância epidemiológica, Incidência, Acidentes

## Abstract

**Objective:**

To estimate the temporal trend of the incidence rates of accidents with venomous animals in children and adolescents in Brazil.

**Methods:**

An ecological time-series study was carried out between 2007 and 2019. Data were obtained from the Brazilian Information System on Diseases of Compulsory Declaration (*Sistema de Informação de Agravos de Notificação* – SINAN). The time series of incidence rates of accidents with venomous animals were stratified by age group (children aged 0 to 9 years and adolescents aged 10 to 19 years), Brazilian macro-regions (North, Northeast, Midwest, Southeast, and South), and type of accident (snake, scorpion, spider, and caterpillar). For trend analysis, the Prais-Winsten model and the Annual Percent Change (APC) were used.

**Results:**

The time series of the incidence rate of accidents with venomous animals in children and adolescents from the North, Northeast, Midwest, and Southeast macro-regions and in children from the South region showed an upward trend. The average annual incidence rates were higher in the age group of 10 to 19 years, except for the South macro-region. Accidents with scorpions, snakes, and spiders, in this order, were the most frequent; the trends in the time series stratified by type of animal varied according to the geographic macro-region.

**Conclusions:**

There was an upward trend in the incidence rate of accidents with venomous animals in children and adolescents in Brazil, except for adolescents in the South macro-region of the country.

## RESUMO

**Objetivo:**

Estimar a tendência temporal das taxas de incidência de acidentes com animais peçonhentos em crianças e adolescentes no Brasil.

**Métodos:**

Foi realizado um estudo ecológico de séries temporais, entre 2007 e 2019. Os dados foram obtidos do Sistema de Informação de Agravos de Notificação (SINAN). As séries temporais das taxas de incidência com animais peçonhentos foram estratificadas por faixa etária (crianças de 0 a 9 anos e adolescentes de 10 a 19 anos), macrorregiões brasileiras (Norte, Nordeste, Centro-Oeste, Sudeste e Sul) e tipo de acidente (serpente, escorpião, aranha e lagarta). Para análise de tendência, foi utilizado o modelo de Prais-Winsten e calculada a variação percentual anual das taxas (*Annual Percentage Change* — APC).

**Resultados:**

As séries históricas da taxa de incidência de acidentes com animais peçonhentos em crianças e adolescentes das macrorregiões Norte, Nordeste, Centro-Oeste e Sudeste e em crianças da macrorregião Sul apresentaram tendência ascendente. As taxas de incidências anuais médias foram maiores na faixa etária de 10 a 19 anos, exceto na macrorregião Sul. Os acidentes com escorpiões, serpentes e aranhas, nesta ordem, foram os mais frequentes; as tendências das séries históricas estratificadas por tipo de animal variaram conforme a macrorregião.

**Conclusões:**

Houve tendência ascendente na taxa de incidência de acidentes com animais peçonhentos em crianças e adolescentes no Brasil, exceto nos adolescentes da macrorregião Sul do país.

## INTRODUCTION

Accidents with venomous animals are those caused by animals that produce toxic substances and provide specific mechanisms for inoculation. This type of accident is frequent and represents a serious public health issue in tropical countries.^1^ In Brazil, accidents involving venomous animals are compulsory notification events since 2010, and those caused by snakes, scorpions, spiders, and caterpillars are of priority interest for the epidemiological surveillance system.^2^ Overall, these accidents are more serious in children than in adults,^3^ considering that the amount of injected venom is independent of the victim's age and the unbound fraction concentration in organs is higher in the pediatric population.

Regarding accidents with snakes, those by snakes from the *Bothrops* genus are the most frequent among snakebites in Brazil. The North and Midwest macro-regions, especially rural areas, account for the highest incidences.^4^ Most accidents are clinically classified as mild; however, the delay in medical assistance and in the beginning of serum therapy can increase the fatality rate.^5^ Conversely, accidents caused by scorpions are predominantly urban, and an increase in cases in the states of the Northeast and Southeast macro-regions has been observed in recent years.^4^ Most cases present a benign clinical course; nevertheless, in the pediatric age group, there is a greater risk for severe clinical manifestations, especially when the accident is caused by the species *T. serrulatus*.^6^

As for spider bites, there are three genera of spiders in Brazil that cause severe symptoms: *Loxosceles* (brown spider), *Phoneutria* (Brazilian wandering spider) and *Latrodectus* (black widow). The occurrence of accidents with each type of spider varies according to geography and time of the year.^4^ In turn, accidents with caterpillars, which are more common in the South and Southeast macro-regions of the country, occur in urban areas, with the exception of lonomic accidents, which are more frequent in rural areas. The severity of the accident is associated with the amount and intensity of contact with the caterpillars, factors that are worthy of attention when dealing with pediatric patients.^7^

In a large and heterogeneous country such as Brazil, regional and temporal variations are expected in the occurrence of accidents with venomous animals. The environmental and habitat transformations of these animals, which result from irregular deforestation, population migration, socioeconomic changes, and diverse climate changes, potentially modify the incidence rates of these accidents.^8^ Thus, periodic epidemiological studies are necessary to know their temporal characteristics and assist in decision-making when developing control and prevention strategies aimed at risk areas and groups. Specifically, ecological time-series studies are an important tool for formulating explanatory hypotheses at the population and regional level, and contribute to the improvement and effectiveness of care. In this context, the objective of this study was to estimate the temporal trend of the incidence rates of accidents with venomous animals in children and adolescents in Brazil.

## METHOD

An ecological time-series study on the incidence of accidents with venomous animals was carried out in children and adolescents residing in Brazilian macro-regions, from 2007 to 2019. All data were obtained from official secondary sources of the Brazilian Ministry of Health, which can be accessed on publicly available websites. Data on reported cases of accidents with venomous animals were obtained from the Brazilian Information System on Diseases of Compulsory Declaration (*Sistema de Informação de Agravos de Notificação –* SINAN)^9^, and the population of each macro-region, according to age group, was obtained from the Brazilian Institute of Geography and Statistics (IBGE).^10^

For the analysis, time series of the annual incidence of accidents with venomous animals were prepared, between the years 2007 and 2019, stratified by age group (children aged 0 to 9 years and adolescents aged 10 to 19 years), Brazilian macro-regions (North, Northeast, Midwest, Southeast and South) and type of accident (snake, scorpion, spider and caterpillar). Incidence rates were estimated by dividing the number of cases by the number of the population and multiplied by 100 thousand, according to the stratification of interest.

For trend analysis, the Prais-Winsten model was used.^11^ The base 10 logarithmic transformation of the incidence was considered as the dependent variable (log[y]) and the centralized-year, as the independent variable (x). Subsequently, the annual percent change (APC) of the incidence and its respective 95% confidence intervals (95%CI) were estimated. The trend was considered statistically significant when zero was not contained in the 95%CI of APC; in this case, the positive APC value represented an upward trend, and the negative APC value, a downward trend. When zero was contained in the 95%CI of APC, the trend was considered stationary. All analyses were performed using the R software version 3.6.3.

According to Resolution No. 466 of 2012, of the National Health Council of the Brazilian Ministry of Health,^12^ this study did not require evaluation by the Research Ethics Committee, as it used secondary, anonymized, and publicly available data.

## RESULTS

Between 2007 and 2019, 597,447 cases of accidents with venomous animals were reported in Brazil in children and adolescents. During this period, there was a 122.5% increase in the annual number of records, from 31,926 cases in 2007 to 71,048 cases in 2019. As for the age group, 55% of the victims aged between 10 and 19 years. Regarding the geographic distribution, 36% of the cases were from the Northeast; 32.5% from the Southeast; 16% from the South; 10.4% from the North; and 5.1% from the Midwest of the country.

**Figure 1:**
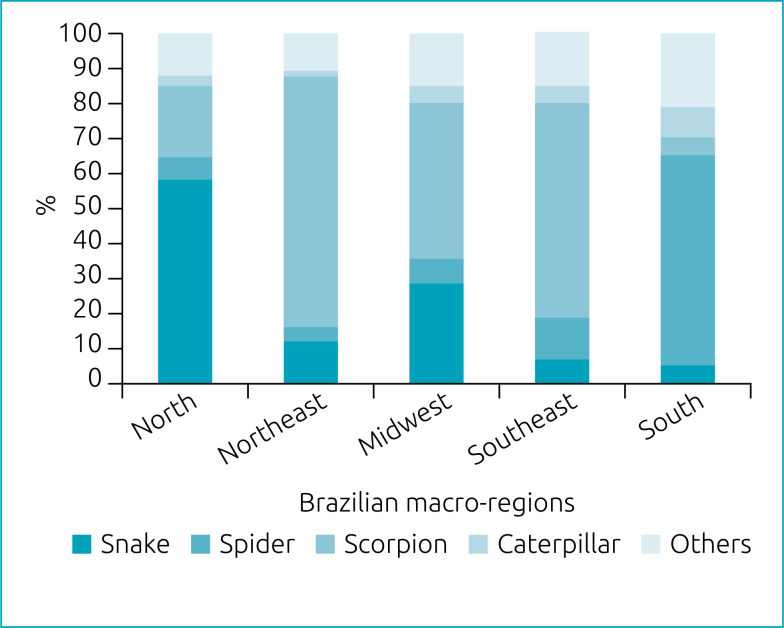
Percent distribution of cases of accidents with venomous animals in children and adolescents according to Brazilian macro-regions and involved animals. Brazil, 2007 to 2019.

Source: Brazilian Ministry of Health.^9,10^

Of the total number of cases in which the involved animal was known (n=580,231; 97.1%), 51% were caused by scorpions; 16.2% by snakes; 14.4% by spiders; 3.3% by caterpillars; and 15% by other animals. Figure 1 shows the percent distribution of cases of accidents with venomous animals according to Brazilian macro-regions and involved animals. There was a predominance of accidents with scorpions in the Northeast, Southeast, and South macro-regions. In the North macro-region, snakebites accounted for almost 60% of the cases. In the South macro-region, spider bite was the accident with the highest percentage of occurrence.

The average annual incidence rates of accidents with venomous animals and the trend analysis, according to macro-region and age group, can be observed in Table 1. The average annual incidence rate during the study period was higher in the age group of 10 to 19 years, except in the South macro-region. The units of analysis showed an upward trend, with the exception of the South macro-region, in the age group of 10 to 19 years, whose time series was stationary.

**Table 1:** Average annual incidence rate and trend analysis of accidents with venomous animals according to macro-region and age group. Brazil, 2007 to 2019.

Macro-region/age group		Average annual incidence rate*	APC	95%CI	Trend
North					
	0 to 9 years	48.83	5.87	4.27–7.50	Upward
	10 to 19 years	87.08	2.53	1.09–4.00	Upward
Northeast					
	0 to 9 years	74.26	12.28	10.23–14.37	Upward
	10 to 19 years	82.44	8.96	6.80–11.17	Upward
Midwest					
	0 to 9 years	40.79	11.85	9.28–14.48	Upward
	10 to 19 years	48.90	8.67	5.77–11.66	Upward
Southeast					
	0 to 9 years	52.60	9.77	8.03–11.53	Upward
	10 to 19 years	62.04	8.36	6.78–9.97	Upward
South					
	0 to 9 years	87.46	2.28	0.73–3.85	Upward
	10 to 19 years	82.85	0.66	-0.06 to 1.39	Stationary

Source: Brazilian Ministry of Health.^9,10^

APC: Annual Percent Change; 95%CI: 95% confidence interval;

* per 100 thousand inhabitants.

Tables 2 and 3 show, respectively, the average annual incidence rates of accidents caused by scorpions, snakes, spiders, and caterpillars, and the trend analysis according to macro-region and age group. The average annual incidence rate of accidents with scorpions was higher in the age group of 10 to 19 years in all Brazilian macro-regions. All analyzed units showed an upward trend. The average annual incidence rate was higher in the Northeast macro-region (Table 2).

**Table 2:** Average annual incidence rate and trend analysis of accidents with scorpions and snakes according to macro-region and age group. Brazil, 2007 to 2019.

Macro-region/age group		Scorpions				Snakes			
		Average annual incidence rate*	APC	95%CI	Trend	Average annual incidence rate*	APC	95%CI	Trend
North									
	0 to 9 years	11.70	8.02	5.74–10.34	Upward	23.22	1.02	-0.45 to 2.51	Stationary
	10 to 19 years	16.85	6.26	4.26–8.31	Upward	57.80	(-0.03)	-1.67 to 1.65	Stationary
Northeast									
	0 to 9 years	55.39	12.74	10.51–15.01	Upward	6.42	1.01	-1.56 to 3.65	Stationary
	10 to 19 years	58.31	10.61	8.41–12.86	Upward	12.98	(-0.85)	-4.03 to 2.43	Stationary
Midwest									
	0 to 9 years	17.60	18.31	15.57–21.12	Upward	9.62	0.98	-0.91 to 2.91	Stationary
	10 to 19 years	22.73	16.14	13.00–19.36	Upward	15.61	(-2.23)	-4.28 to −0.13	Downward
Southeast									
	0 to 9 years	26.16	11.99	9.38–14.66	Upward	3.45	(-2.08)	-4.01 to −0.11	Downward
	10 to 19 years	35.02	11.90	9.99–13.84	Upward	6.66	(-3.71)	-5.71 to −1.66	Downward
South									
	0 to 9 years	3.10	15.42	10.79–20.25	Upward	3.94	(-2.55)	-4.1 to −0.98	Downward
	10 to 19 years	5.31	11.59	8.94–14.31	Upward	7.42	(-5.17)	-6.22 to −4.1	Downward

Source: Brazilian Ministry of Health.^9,10^

APC: Annual Percent Change; 95%CI: 95% confidence interval;

* per 100 thousand inhabitants.

**Table 3:** Average annual incidence rate and trend analysis of accidents with spiders and caterpillars according to macro-region and age group. Brazil, 2007 to 2019.

Macro-region/age group		Spiders				Caterpillars			
		Average annual incidence rate*	APC	95%CI	Trend	Average annual incidence rate*	APC	95%CI	Trend
North									
	0 to 9 years	2.59	9.21	6.88–11.6	Upward	2.17	8.32	0.19–17.1	Upward
	10 to 19 years	2.99	5.48	2.82–8.21	Upward	1.06	9.74	0.65–19.65	Upward
Northeast									
	0 to 9 years	1.58	13.41	10.49–16.41	Upward	0.77	15.09	10.65–19.7	Upward
	10 to 19 years	1.81	12.92	9.91–16.01	Upward	0.51	10.12	5.89–14.52	Upward
Midwest									
	0 to 9 years	2.71	12.56	8.87–16.38	Upward	1.78	14.7	5.29–24.96	Upward
	10 to 19 years	2.82	10.85	6.73–15.13	Upward	0.78	17.81	8.41–28.02	Upward
Southeast									
	0 to 9 years	6.00	8.52	6.86–10.2	Upward	2.30	4.92	-0.26 to 10.38	Stationary
	10 to 19 years	7.00	6.33	4.58–8.12	Upward	1.60	3.44	-1.17 to 8.26	Stationary
South									
	0 to 9 years	47.94	1.5	0.03–2.98	Upward	9.15	0.48	-4.89 to 6.16	Stationary
	10 to 19 years	46.86	(-0.08)	-0.52 to 0.36	Stationary	6.70	(-3.25)	-7.26 to 0.94	Stationary

Source: Brazilian Ministry of Health.^9,10^

APC: Annual Percent Change; 95%CI: 95% confidence interval;

* per 100 thousand inhabitants.

Regarding snakebites, the North macro-region had the highest incidence rates in both age groups analyzed. The average annual incidence rate was higher in the age group of 10 to 19 years in all Brazilian macro-regions. The time series of the North and Northeast were stationary, whereas those of the Southeast and South showed a downward trend in the incidence of this type of accident; in the Midwest, a mixed pattern was observed (Table 2).

As for accidents with spiders, the average annual incidence rate during the study period was higher in the age group of 10 to 19 years, except in the South macro-region. The units of analysis showed an upward trend, with the exception of the South macro-region, in the age group of 10 to 19 years, whose time series was stationary. The average annual incidence rate was significantly higher in the South macro-region (Table 3).

Finally, the average annual incidence rate of accidents with caterpillars was higher in the age group of 0 to 9 years in all macro-regions. North, Northeast, and Midwest showed an upward trend, whereas in the Southeast and South stationary time series were observed. The average annual incidence rate was higher in the South macro-region for both age groups (Table 3).

## DISCUSSION

The present study verified an upward trend in the incidence rate of accidents with venomous animals in children and adolescents in Brazil, except for adolescents in the South macro-region. The average annual incidence rates were higher in the age group of 10 to 19 years, except for the South macro-region. Accidents with scorpions, snakes, and spiders, in this order, were the most frequent in this population; incidence and distribution rates varied according to the geographic area of the country.

In Brazil, there has been a progressive improvement in the registration of diseases of compulsory notification in recent decades. Thus, the gradual increase in the incidence rate of accidents with venomous animals during the analyzed period may reflect the increase in notifications by epidemiological surveillance services, without necessarily having had a real increase in the number of cases in the population of children and teenagers. Nevertheless, the environmental imbalance with extensive areas of irregular deforestation and the acceleration of the urbanization process, which several Brazilian cities have undergone in recent decades, are factors that may be related to the increase in the number of cases.^8^ In addition, stationary time series and those with a downward trend were verified, such as those observed in accidents with snakes, which reinforces the idea that, in fact, there is an increase in the number of accidents with venomous animals, especially those caused by scorpions.

A previous study on accidents with venomous animals in the Brazilian population, which analyzed the period from 2001 to 2012, showed that most accidents were caused by scorpions, snakes, and spiders, whose occurrence showed great regional variations.^13^ During this period, the constant numerical increase in cases caused by all animal species was attributed to several factors, including the increase in the human population, the gradual improvement of the epidemiological surveillance system, and, probably, environmental and socioeconomic factors differently affecting the incidence of accidents per each zoological group and region..^13^

Regarding snakebites, the estimated trends were stationary or downward in children and adolescents residing in Brazilian macro-regions. Matos et al.^14^, by analyzing the period from 2003 to 2012, had already found a stationary trend in the historical series for the country altogether.

The highest incidence rates of accidents with caterpillars have been traditionally found in the South and Southeast of Brazil.^15^ However, in the present study, this indicator showed to be increasing in the North, Northeast, and Midwest macro-regions, which raises an alert for the possible emerging nature of this type of accident, already evidenced in other countries.^16^

As limitations of this study, the authors highlight the use of secondary data, which involves the quality of records and is directly associated with the organization of health services in each location. About 86% of the reports of snakebites and 90% of the reports of spider bites presented ignored and/or unfilled information in the field concerning the genus of the involved animal, considering the age group of 0 to 19 years, in the period from 2007 to 2019, which made the analysis unfeasible. This is an essential piece of information to support preventive and health promotion actions, considering that there are specificities in the habits of different types of animals, in addition to providing indications that help in the therapeutic conduct according to the region where the accident occurred.

Another point worth reflecting on is the more urban nature of most scorpion accidents,^17^ which may contribute to the increase in notifications, at the expense of snakebites, for example, which are more common in rural regions and away from urban centers and their health services.

It is also worth mentioning that, although ecological studies are useful to formulate hypotheses, it is necessary to point out the main limitation of this study design: the ecological bias, which consists of attributing inferences about individuals based on aggregated data from groups. In this study, the authors carefully used smaller units of analysis, stratifying the study population according to age group and Brazilian macro-region. Thus, it is believed that the groups became more homogeneous in relation to exposures, contributing to the minimization of this type of bias.^18^ Finally, the stratification of the data also allowed the authors to use the crude incidence rates of accidents with venomous animals rather than the standardized incidence rate.

Conversely, unlike other Brazilian studies that analyzed specific locations,^3,8,19^ the present study used data from the entire national territory, collected over a long period. In view of the vast geographic area and population size of Brazil, an ecological approach potentially and quickly identifies the existence of vulnerable groups, which require priority actions.^20^

Although there is an upward trend in the incidence of accidents by venomous animals in children and adolescents in most regions of Brazil, these diseases can be prevented through educational actions and health policies aimed at protecting this population. Furthermore, the improvement of strategies to ensure diagnosis and institution of early treatment is necessary to reduce the severity and, consequently, the number of fatal outcomes associated with these diseases. Within this context, the authors believe that this study contributed with updated and substantiated information about accidents by venomous animals in children and adolescents in Brazil.

## References

[1] Braga JR, Souza MM, Melo IM, Faria LE, Jorge RJ (2021). Epidemiology of accidents involving venomous animals in the State of Ceará, Brazil (2007-2019). Rev Soc Bras Med Trop.

[2] Brazil – Ministério da Saúde (2010). Define as terminologias adotadas em legislação nacional, conforme disposto no Regulamento Sanitário Internacional 2005 (RSI 2005), a relação de doenças, agravos e eventos em saúde pública de notificação compulsória em todo o território nacional e estabelecer fluxo, critérios, responsabilidades e atribuições aos profissionais e serviços de saúde.

[3] Tavares AV, Araújo KA, Marques MR, Leite R (2020). Epidemiology of the injury with venomous animals in the state of Rio Grande do Norte, Northeast of Brazil. Cienc Saude Coletiva.

[4] Brazil – Ministério da Saúde (2019). Secretaria de Vigilância em Saúde. Guia de Vigilância em Saúde.

[5] Brenes-Chacón H, María Gutiérrez J, Camacho-Badilla K, Soriano-Fallas A, Ulloa-Gutierrez R, Valverde-Muñoz K (2019). Snakebite envenoming in children: a neglected tropical disease in a Costa Rican pediatric tertiary care center. Acta Trop.

[6] Albuquerque CM, Santana PL, Amorim ML, Pires SC (2013). Pediatric epidemiological aspects of scorpionism and report on fatal cases from *Tityus stigmurus* stings (Scorpiones: Buthidae) in State of Pernambuco, Brazil. Rev Soc Bras Med Trop.

[7] Rubio GB (2001). Epidemiological surveillance of distribution of the caterpillar Lonomia obliqua Walker, 1855, in the State of Paraná, Brazil. Cad Saude Publica.

[8] Oliveira NR, Sousa AN, Belmino JF, Furtado SS, Leite RS (2015). The epidemiology of envenomation via snakebite in the State of Piauí, Northeastern Brazil. Rev Soc Bras Med Trop.

[9] Brazil – Ministério da Saúde [homepage on the Internet] Banco de dados do Sistema Único de Saúde - DATASUS - Informações de Saúde: Epidemiológicas e Morbidade.

[10] Brazil – Ministério da Saúde[homepage on the Internet] Banco de dados do Sistema Único de Saúde - DATASUS - Informações de Saúde: Demográfica e Socioeconômicas.

[11] Antunes JL, Cardoso MR (2015). Using time series analysis in epidemiological studies. Epidemiol Serv Saúde.

[12] Brazil - Ministério da Saúde (2012). Resolução n°.466 de 12 de dezembro de 2012.

[13] Chippaux JP (2015). Epidemiology of envenomations by terrestrial venomous animals in Brazil based on case reporting: from obvious facts to contingencies. J Venom Anim Toxins Incl Trop Dis.

[14] Matos RR, Ignotti E (2020). Incidence of venomous snakebite accidents by snake species in Brazilian biomes. Cienc Saude Coletiva.

[15] Favalesso MM, Cuervo PF, Casafús MG, Guimarães AT, Peichoto ME (2021). *Lonomia* envenomation in Brazil: an epidemiological overview for the period 2007–2018. Trans R Soc Trop Med Hyg.

[16] Sánchez MN, Chagas MA, Casertano SA, Cavagnaro LE, Peichoto ME (2015). Accidentes causados por la oruga Lonomia obliqua (Walker,1855). Un problema emergente. Medicina (Buenos Aires).

[17] Brazil - Ministério da Saúde (2009). Manual de Controle de Escorpiões.

[18] Rothman KJ, Greenland S, Lash TL (2011). Epidemiologia moderna.

[19] Taniele-Silva J, Martins LG, Sousa MB, Souza LM, Cardoso RM, Velasco SR (2020). Retrospective clinical and epidemiological analysis of scorpionism at a referral hospital for the treatment of accidents by venomous animals in Alagoas State, Northeast Brazil, 2007-2017. Rev Inst Med Trop São Paulo.

[20] Machado C (2016). Um panorama dos acidentes por animais peçonhentos no Brasil. J Health NPEPS.

